# Morphometric and Statistical Analysis of Pollen Morphology in Seven Woody Species of Betulaceae

**DOI:** 10.3390/plants15060947

**Published:** 2026-03-19

**Authors:** Hülya Caner, Rüya Yılmaz Dağdeviren, Nurgül Karlıoğlu Kılıç, Gülan Güngör

**Affiliations:** 1 Department of Marine Geology and Geophysics, Institute of Marine Sciences and Management, Istanbul University, 34000 Istanbul, Türkiye; 2 Department of Forest Botany, Faculty of Forestry, Istanbul University-Cerrahpaşa, 34473 Istanbul, Türkiye; ruya.dagdeviren@iuc.edu.tr (R.Y.D.); nurgulk@iuc.edu.tr (N.K.K.); 3 Institute of Social Sciences, Istanbul University, 34000 Istanbul, Türkiye; gulangungor3636@gmail.com

**Keywords:** pollen morphology, Betulaceae, *Carpinus betulus*, *Alnus glutinosa*, PCA

## Abstract

Morphological characteristics of pollen grains, including shape, size, pore number, and exine thickness, vary significantly among species and enable the reliable use of palynological data in taxonomic studies. In this context, the present study investigates the pollen morphology of seven Betulaceae taxa (*Alnus glutinosa*, *Betula pendula*, *Carpinus betulus*, *Carpinus orientalis*, *Corylus avellana*, *Corylus colurna*, and *Ostrya carpinifolia*). Detailed morphometric measurements were carried out using Light Microscopy (LM), and high-resolution images were obtained using Scanning Electron Microscopy (SEM). For each taxon, thirty measurements were taken for the main pollen characters, including polar axis length (P), equatorial diameter (E), pore length (plg), pore width (plt), and exine thickness (Ex). Interspecific differences were evaluated using one-way ANOVA, Tukey’s HSD test, and Principal Component Analysis (PCA), and a diagnostic pollen identification key was developed for the investigated species. The results demonstrate statistically significant interspecific variation in pollen size, pore characteristics, and exine thickness. In the PCA ordination, the first principal component (PC1) was mainly associated with pollen size (P and E), clearly separating *Carpinus betulus* from the remaining taxa. The second principal component (PC2) was primarily related to pore length (plg) and contributed to the separation of *Alnus glutinosa* from the other small-pollen species. These results show that quantitative pollen morphological characters provide reliable criteria for distinguishing closely related Betulaceae taxa.

## 1. Introduction

Pollen morphology is a sub-discipline of palynology that encompasses the examination of morphological characteristics of pollen grains, including their shape, size, exine structure, aperture type, and surface ornamentation. These features provide essential information for the systematic classification of plants, the clarification of taxonomic relationships, and the interpretation of evolutionary processes [1,2,3]. Statistically significant morphological differences among taxa enable the reliable use of palynological data in taxonomic studies. Moreover, pollen morphology plays a crucial role in plant systematics by revealing phylogenetic relationships among taxa and in paleopalynological studies by facilitating the reconstruction of past plant assemblages and their spatial distributions [1,4]. In this context, the detailed investigation of pollen morphology in selected taxa of the Betulaceae constitutes one of the main objectives of the present study.

Betulaceae is represented worldwide by two subfamilies, Betuloideae and Coryloideae, including approximately 205 species distributed mainly in the Northern Hemisphere. The investigated taxa belong to these two subfamilies and exhibits distinct geographical distributions. The taxonomic position and geographical distribution of the studied species are summarized in Table 1.

The first study on pollen morphology of the Betulaceae was conducted by Wodehouse in 1935 [8]. This study demonstrated that pollen sizes of Betulaceae taxa range between 20 and 40 µm and that the number of pores varies from 3 to 7. In his study on the pollen morphology of the Betulaceae, Erdtman [1] examined approximately 40 species belonging to six genera. He reported that the number of pores in this family ranges from 3 to 7 and that the pollen grains exhibit an aspidote structure. He also noted that the equatorial diameters of the pollen grains range between 20 and 30 µm. Furthermore, Erdtman [1] described the presence of arc-like structures extending from pore to pore in the pollen grains of taxa belonging to the genus *Alnus*. Kuprianova [9], in her study on Betulaceae pollen, divided the taxa within the family into three subfamilies. According to this classification, *Alnus* and *Betula* were placed in the subfamily Betuleae, *Corylus* in Coryleae, and *Carpinus* and *Ostrya* in Carpineae. Praglowski [10] reported that Betulaceae pollen grains are isopolar and aspidote, with pollen sizes ranging from 15 to 40 µm, in a pollen morphological study of certain trees and shrubs in Sweden. Aytuğ et al. [11], in his investigation of pollen from various plant species in Istanbul, examined five species belonging to the Betulaceae, measuring pollen type, aperture number, polar axis, equatorial diameter, and exine thickness, and provided a concise description of their pollen morphology. Blackmore et al. [12] conducted a detailed study on the pollen morphology of Betulaceae taxa and developed a comprehensive pollen identification key for the investigated species. Similarly, Alan [13], in his study on pollen morphology of Betulaceae taxa, reported that pollen shape varies from suboblate to spheroidal and also constructed a pollen identification key for the studied species.

Numerous palynological studies have documented pollen morphological characteristics of Betulaceae taxa, including pollen size, aperture configuration, and exine thickness (Table 2).

Although numerous studies have been conducted on pollen morphology within the Betulaceae, investigations in which the obtained measurements are tested using statistical analyses remain limited. In this context, detailed morphological measurements of pollen grains from *Alnus glutinosa*, *Betula pendula*, *Carpinus betulus*, *Carpinus orientalis*, *Corylus avellana*, *Corylus colurna*, and *Ostrya carpinifolia* were carried out using light microscopy, and high-resolution images were additionally obtained using scanning electron microscopy (SEM). To reveal interspecific differences, statistical evaluations were performed using one-way ANOVA (ANalysis Of Variance), Tukey’s HSD test (Tukey’s Honestly Significant Difference) and Principal Component Analysis (PCA). Furthermore, a diagnostic key highlighting species-specific differences in pollen morphology was developed.

## 2. Results

### 2.1. Palinological Results

**Figure 1:**
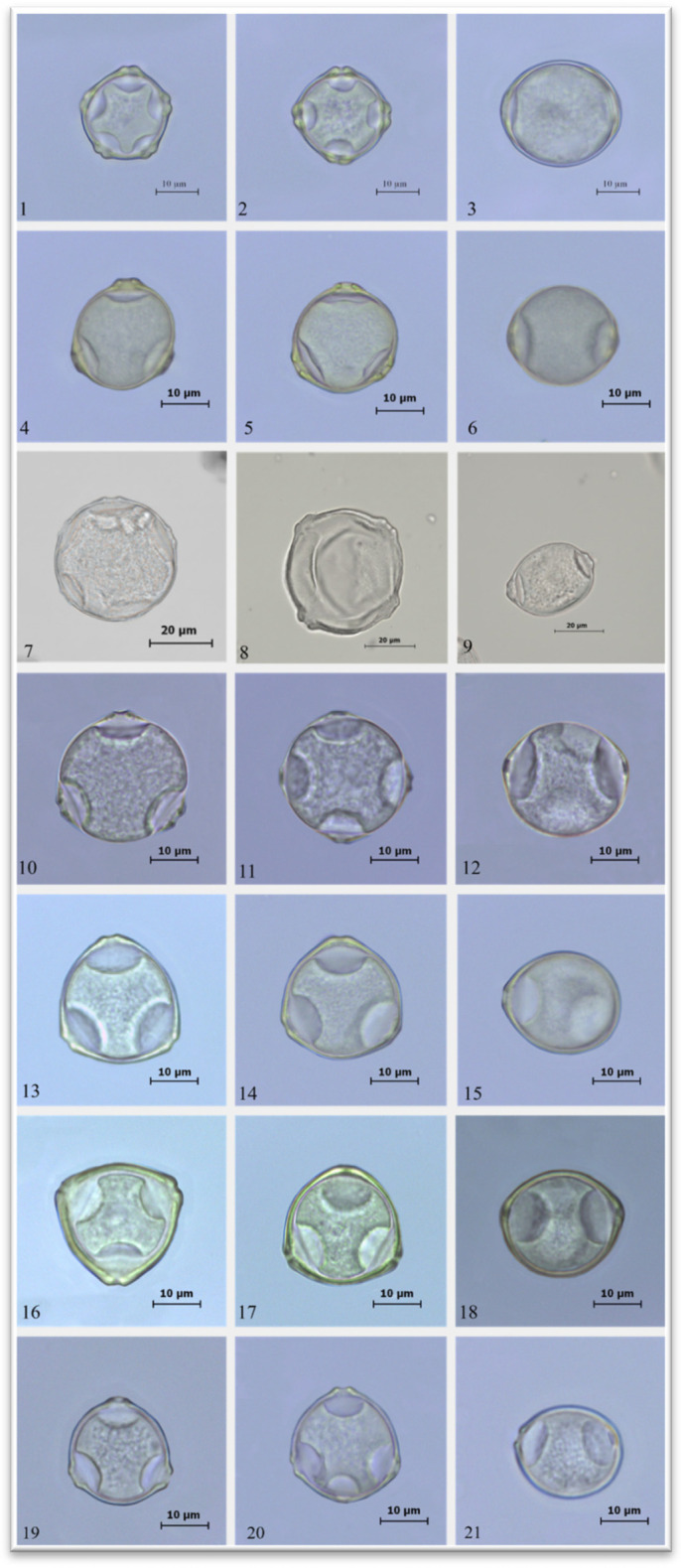
Polar and equatorial views of pollen grains under LM. (**1**,**2**) Polar view of *Alnus glutinosa* pollen grains, (**3**) equatorial view of *Alnus glutinosa* pollen grains, (**4**,**5**) polar view of *Betula pendula* pollen grains, (**6**) equatorial view of *Betula pendula* pollen grains, (**7**,**8**) polar view of *Carpinus betulus* pollen grains, (**9**) equatorial view of *Carpinus betulus* pollen grains, (**10**,**11**) polar view of *Carpinus orientalis* pollen grains, (**12**) equatorial view of *Carpinus orientalis* pollen grains, (**13**,**14**) polar view of *Corylus avellana* pollen grains, (**15**) equatorial view of *Corylus avellana* pollen grains, (**16**,**17**) polar view of *Corylus colurna* pollen grains, (**18**) equatorial view of *Corylus colurna* pollen grains, (**19**,**20**) polar view of *Ostrya carpinifolia* pollen grains, (**21**) equatorial view of *Ostrya carpinifolia* pollen grains.

**Figure 2:**
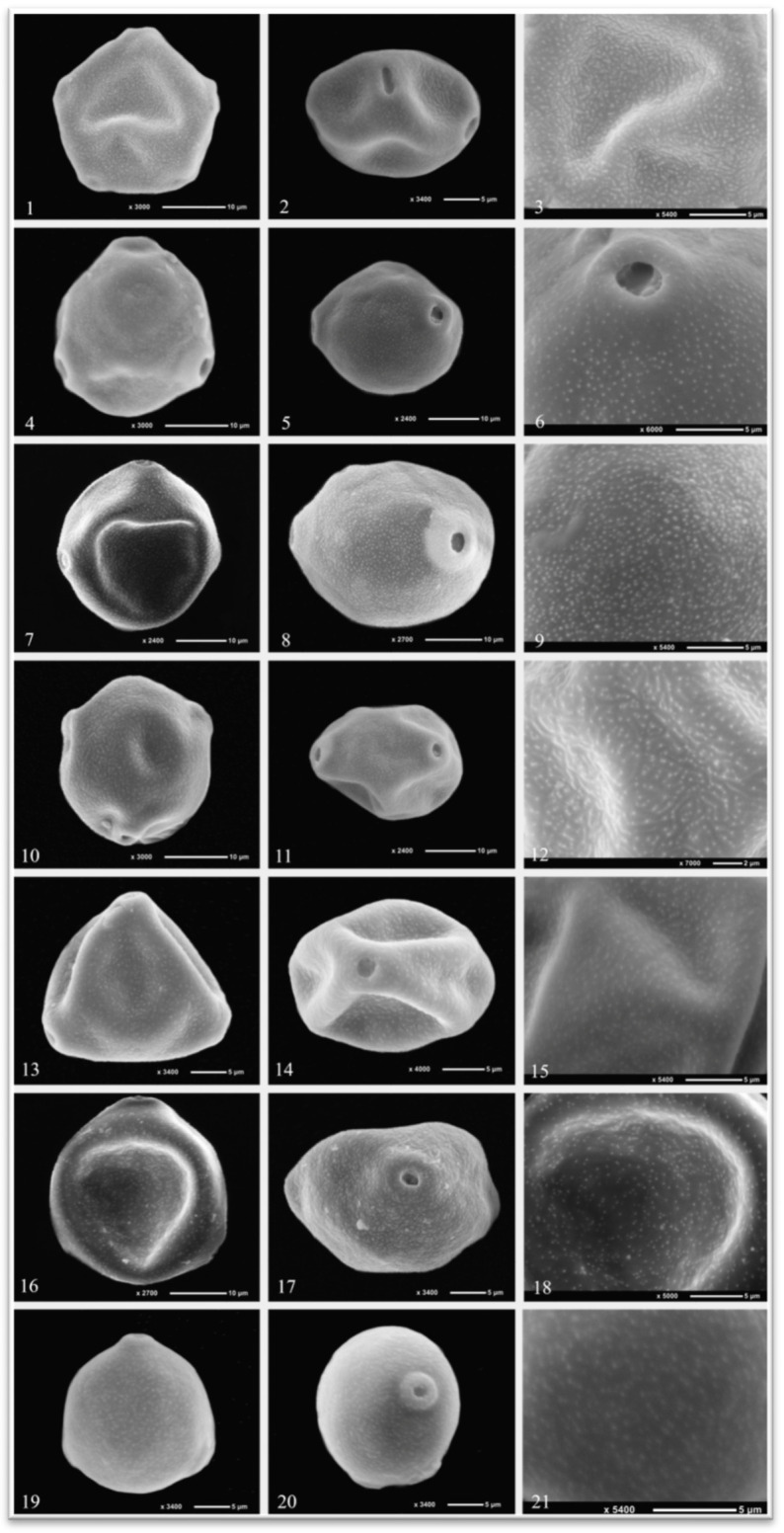
Polar and equatorial views of pollen grains under SEM. (**1**,**2**) Polar view of *Alnus glutinosa* pollen grains, (**3**) equatorial view of *Alnus glutinosa* pollen grains, (**4**,**5**) polar view of *Betula pendula* pollen grains, (**6**) equatorial view of *Betula pendula* pollen grains, (**7**,**8**) polar view of *Carpinus betulus* pollen grains, (**9**) equatorial view of *Carpinus betulus* pollen grains, (**10**,**11**) polar view of *Carpinus orientalis* pollen grains, (**12**) equatorial view of *Carpinus orientalis* pollen grains, (**13**,**14**) polar view of *Corylus avellana* pollen grains, (**15**) equatorial view of *Corylus avellana* pollen grains, (**16**,**17**) polar view of *Corylus colurna* pollen grains, (**18**) equatorial view of *Corylus colurna* pollen grains, (**19**,**20**) polar view of *Ostrya carpinifolia* pollen grains, (**21**) equatorial view of *Ostrya carpinifolia* pollen grains.

The pollen grains of *Alnus glutinosa* possess a five-porate (stephanoporate) apertur type (Figure 1 and Figure 2). The length of the polar axis (P) ranges from 17.53 to 24.71 µm, while the equatorial diameter (E) varies between 20.96 and 29.50 µm. The P/E ratio is 0.88, indicating a suboblate pollen shape according to the classification of Erdtman [1]. The pore length (plg) ranges from 3.05 to 4.22 µm, and the pore width (plt) from 1.27 to 2.85 µm. The exine thickness varies between 1.03 and 1.65 µm (Table 3).

The pollen grains of *Betula pendula* are characterized by a triporate aperture structure (Figure 1 and Figure 2). The polar axis length (P) ranges from 20.64 to 24.82 µm, while the equatorial diameter (E) varies between 23.27 and 26.96 µm. The P/E ratio is 0.89, and according to Erdtman [1], the pollen shape is oblate-spheroidal. The pore length (plg) ranges from 2.58 to 3.51 µm, and the pore width (plt) from 2.03 to 3.24 µm. The exine thickness was determined to range between 0.63 and 1.16 µm (Table 3).

In *Carpinus betulus*, 40% of the pollen grains display a four-porate (tetraporate) aperture type, while the remaining 60% are characterized by a five-porate (stephanoporate) aperture structure (Figure 1 and Figure 2). The polar axis length (P) ranges from 28.52 to 33.72 µm, while the equatorial diameter (E) varies between 34.42 and 39.67 µm. The P/E ratio is 0.86, indicating a suboblate pollen shape according to the classification of Erdtman [1]. The pore length (plg) ranges from 2.86 to 4.33 µm, and the pore width (plt) from 2.32 to 3.72 µm. The exine thickness varies between 0.84 and 1.38 µm (Table 3).

The pollen grains of *Carpinus orientalis* are triporate in aperture type (Figure 1 and Figure 2). The polar axis length (P) ranges from 25.53 to 29.40 µm, and the equatorial diameter (E) from 30.00 to 33.91 µm. The P/E ratio is 0.88, and according to Erdtman [1], the pollen shape is classified as suboblate. The pore length (plg) ranges from 2.07 to 2.98 µm, while the pore width (plt) varies between 1.57 and 2.36 µm. The exine thickness was measured to range from 0.72 to 1.18 µm (Table 3).

The pollen grains of *Corylus avellana* are characterized by a triporate aperture type (Figure 1 and Figure 2). The polar axis length (P) ranges from 20.71 to 23.70 µm, while the equatorial diameter (E) varies between 25.26 and 30.90 µm. The P/E ratio is 0.83, indicating a suboblate pollen shape according to the classification of Erdtman [1]. The pore length (plg) ranges from 2.08 to 2.95 µm, and the pore width (plt) from 1.82 to 2.71 µm. The exine thickness varies between 1.06 and 1.53 µm (Table 3).

The pollen grains of *Corylus colurna* also exhibit a triporate aperture structure (Figure 1 and Figure 2). The polar axis length (P) ranges from 26.01 to 29.33 µm, while the equatorial diameter (E) varies between 27.80 and 32.46 µm. The P/E ratio is 0.93, and based on Erdtman’s [1] classification, the pollen shape is oblate-spheroidal. The pore length (plg) ranges from 2.36 to 3.76 µm, and the pore width (plt) from 2.11 to 3.25 µm. The exine thickness was determined to range between 1.09 and 1.92 µm (Table 3).

The pollen grains of *Ostrya carpinifolia* are triporate in aperture type (Figure 1 and Figure 2). The polar axis length (P) ranges from 21.09 to 23.92 µm, whereas the equatorial diameter (E) varies between 22.71 and 26.91 µm. The P/E ratio is 0.91, indicating an oblate-spheroidal pollen shape according to Erdtman [1]. The pore length (plg) ranges from 2.26 to 3.49 µm, and the pore width (plt) from 2.01 to 2.96 µm. The exine thickness was measured to range between 0.89 and 1.52 µm (Table 3).

In this study, the pollen ornamentation of the seven investigated species was identified as psilate when examined under light microscopy, whereas observations using scanning electron microscopy revealed a microechinate ornamentation pattern (Figure 1 and Figure 2).

### 2.2. Statistical Results

The measured pollen morphological characters (P, E, plg, plt, and Ex) of seven species belonging to the Betulaceae were statistically evaluated in this study. Descriptive statistics (mean and standard deviation) were calculated for each species and each variable in order to assess intraspecific variation (Table 4).

The results of the one-way ANOVA revealed highly significant differences among species for all measured variables (Table 5; *p* < 0.001). These findings indicate that pollen morphology possesses a strong discriminatory power at the species level.

Pollen size, expressed as polar axis length (P) and equatorial diameter (E), emerged as the most prominent morphological variables explaining interspecific differences. According to the Tukey HSD test, *Carpinus betulus* exhibited significantly higher values for both pollen length and width compared to all other species, forming a distinct group within the Betulaceae family. *Carpinus orientalis* and *Corylus colurna* clustered together with intermediate pollen dimensions, whereas *Alnus glutinosa*, *Betula pendula*, *Corylus avellana*, and *Ostrya carpinifolia* were classified as small-pollen species. No significant differences were detected in most pairwise comparisons among these four species. These results indicate that P and E are key discriminative variables for distinguishing Betulaceae species.

Analyses of pore length (plg) revealed significant differences among species (Table 5; *p* < 0.001). The highest plg values were recorded in *Alnus glutinosa* and *Carpinus betulus*, distinguishing these taxa from the remaining species based on pore morphology. *Betula pendula* and *Ostrya carpinifolia* exhibited intermediate pore lengths, whereas *Carpinus orientalis*, *Corylus avellana*, and *Corylus colurna* showed significantly lower values.

With respect to pore width (plt), the highest mean values were recorded in *Carpinus betulus*. *Betula pendula*, *Corylus colurna* and *Ostrya carpinifolia* exhibited pores of intermediate width, whereas *Alnus glutinosa*, *Carpinus orientalis* and *Corylus avellana* were characterized by relatively narrower pore structures. Exine thickness showed marked variation among species. *Corylus colurna*, and *C. avellana* exhibited the thickest exine within the family and formed a distinct morphological group. *Alnus glutinosa* and *Ostrya carpinifolia* were characterized by intermediate exine thickness values, while *Betula pendula*, *Carpinus orientalis*, and *Carpinus betulus* possessed the thinnest exine.

**Figure 3:**
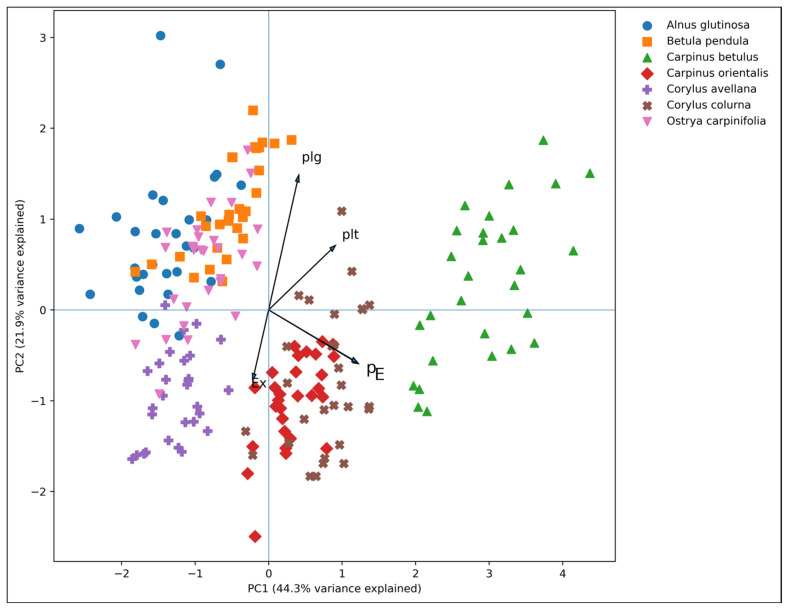
The results indicate that pollen morphological characters within the Betulaceae exhibit clear and statistically significant differences among species. When pollen size, pore characteristics, and exine thickness are evaluated together, the species can be reliably distinguished based on their pollen morphology.

PCA, conducted to evaluate the combined effect of the five morphological variables, demonstrated a clear separation of species within the multivariate morphological space. The first two principal components accounted for 66.1% of the total variance, with PC1 explaining 44.3% and PC2 explaining 21.9%. The first principal component (PC1) was primarily associated with pollen size variables (P and E) and pore width (plt), among which *Carpinus betulus* was clearly separated from the remaining species. The second principal component (PC2) was mainly related to pore length (plg) and particularly contributed to the separation of *Alnus glutinosa* from the other small-pollen species (Figure 3).

In the PCA ordination, *Corylus* species were positioned close to each other in relation to exine thickness, whereas the small-pollen species (*Alnus glutinosa*, *Betula pendula*, *Corylus avellana,* and *Ostrya carpinifolia*) generally clustered at low PC1 values (Figure 3).

All investigated taxa share similar general ornamentation patterns, appearing psilate under LM and microechinate under SEM. Consequently, species-level discrimination does not rely on exine sculpture type but primarily on pore number and quantitative morphometric parameters. The first and most decisive diagnostic criterion is pore number. *Alnus glutinosa* is clearly distinguished by its predominantly 5-porate pollen grains, whereas *Carpinus betulus* differs from all other taxa by exhibiting 4–5-porate pollen in combination with markedly larger pollen dimensions (P ≈ 31 µm; E ≈ 36 µm). The remaining taxa possess strictly 3-porate pollen grains. Within the 3-porate group, differentiation is achieved through polar axis length and exine thickness. *Corylus colurna* is characterized by relatively large pollen grains (P ≈ 28 µm) and the thickest exine (≈1.54 µm) among the studied taxa. *Carpinus orientalis* shows slightly smaller pollen grains (P ≈ 27 µm) and shorter pore length (≈2.59 µm). Species with smaller pollen grains (P < 27 µm) are further distinguished by exine thickness. *Betula pendula* is identified by its notably thin exine (≈ 0.86 µm), whereas *Corylus avellana* (≈1.32 µm) and *Ostrya carpinifolia* (≈1.21 µm) possess thicker exines; these latter two taxa are additionally separated by equatorial diameter values, with *C. avellana* exhibiting slightly larger E values (≈27 µm) than *O. carpinifolia* (≈25 µm).

This hierarchically structured diagnostic key is not merely descriptive but statistically supported. The present study establishes a quantitatively validated and conceptually structured framework for interpreting pollen morphological differentiation within Betulaceae (Table 6).

## 3. Discussion

The diagnostic key presented in the Results section demonstrates that species-level discrimination within the investigated Betulaceae taxa follows a clear hierarchical structure based on pore number, pollen size, and exine thickness. In particular, pore number represents the primary diagnostic character separating *Alnus glutinosa* and *Carpinus betulus* from the remaining taxa, while pollen size and exine thickness provide additional resolution among the strictly triporate species. This hierarchical organization of pollen characters is supported by the statistical analyses performed in this study and indicates that morphometric pollen traits constitute reliable diagnostic criteria for distinguishing closely related Betulaceae taxa.

The present study establishes a quantitatively validated and conceptually structured framework for interpreting pollen morphological differentiation within Betulaceae. Although previous palynological investigations [1,8,10,12] provided detailed descriptive accounts of pollen size ranges, aperture configurations, and ornamentation patterns, they largely relied on qualitative comparisons. In contrast, the integration of precise morphometric measurements with rigorous univariate (ANOVA, Tukey’s HSD test) and multivariate (PCA) statistical analyses demonstrates that interspecific variation is hierarchically organized and statistically robust.

Pollen size (polar axis and equatorial diameter) emerged as the primary axis of morphological differentiation. The exceptionally high ANOVA F-values for P (274.74; *p* < 0.001) and E (283.16; *p* < 0.001) indicate that interspecific variance substantially exceeds intraspecific variability, quantitatively confirming earlier qualitative observations that pollen size represents a key taxonomic character within the family [1,12]. The distinct separation of *Carpinus betulus* as a large-pollen taxon aligns with previous reports [10,13], while *Alnus glutinosa*, *Betula pendula*, *Corylus avellana*, and *Ostrya carpinifolia* formed a small-pollen group. *Carpinus orientalis* and *Corylus colurna* occupied intermediate morphometric positions. This size-based organization reflects genus-level differentiation patterns between Betuloideae and Coryloideae [9,12]. PCA independently confirmed this hierarchy, with pollen size dominating PC1 and accounting for the largest proportion of total variance.

Aperture morphology contributed to secondary differentiation among taxa. The predominance of stephanoporate pollen in *Alnus glutinosa* confirms its generic distinctiveness [1], whereas the triporate configuration observed in Coryloideae taxa supports established generic boundaries [12]. Significant interspecific differences in pore length (plg; F = 42.02, *p* < 0.001) and pore width (plt; F = 45.59, *p* < 0.001) demonstrate that aperture dimensions enhance taxonomic resolution. PCA further indicated that pore length (plg) contributed primarily to PC2, suggesting that aperture traits function as fine-scale discriminators rather than primary structuring variables.

Exine thickness also showed significant interspecific variation (F = 67.91, *p* < 0.001). Although not sufficient alone for complete species separation, its integration with pollen size and aperture traits enhances diagnostic reliability and supports a multi-character evaluation approach.

The morphometric differentiation observed among the investigated taxa also reflects broader phylogenetic patterns within Betulaceae. In particular, the separation of *Alnus* and *Betula* from the remaining genera corresponds well with the traditional subdivision of the family into the subfamilies Betuloideae and Coryloideae [9,12]. Molecular phylogenetic studies based on nuclear and plastid DNA sequences likewise support this evolutionary division, identifying these two subfamilies as well-defined clades within Betulaceae [29,30]. The morphometric differences documented in the present study—especially in pollen size, pore number, and exine thickness—therefore appear to reflect not only species-level variation but also deeper evolutionary differentiation within the family. In this context, quantitative pollen morphological characters provide complementary evidence for interpreting phylogenetic relationships within Betulaceae.

Despite the robust morphometric and statistical framework applied in this study, there are certain limitations that should be acknowledged. The analysed material represents specimens collected from limited geographical localities within Türkiye and may therefore not fully capture the entire range of intraspecific morphological variability across the broader distribution of these taxa. Future studies incorporating samples from wider geographical regions and larger population sizes could provide a more comprehensive evaluation of pollen morphological variability and further refine the taxonomic significance of these characters within Betulaceae.

Crucially, the concordance between ANOVA, Tukey’s HSD test, and PCA demonstrates that pollen morphology in Betulaceae is not only descriptively distinct but also statistically robust. By transforming classical descriptive palynology into a quantitatively testable and hierarchically structured morphometric framework, this study introduces a reproducible model for species-level discrimination. This statistically supported diagnostic hierarchy reduces interpretative subjectivity—historically complicated by morphological overlap among closely related taxa—and strengthens the application of pollen morphology in systematic taxonomy and Quaternary paleoenvironmental reconstruction. Furthermore, the proposed framework offers a transferable analytical template for other wind-pollinated woody plant families.

Beyond its taxonomic implications, the statistically supported morphometric framework developed in this study provides a reproducible approach for pollen-based species discrimination within wind-pollinated woody taxa. By integrating quantitative measurements with multivariate statistical analyses, the study demonstrates that pollen morphology can serve not only as a descriptive tool but also as a robust analytical proxy for systematic and paleoenvironmental research. Consequently, this framework may contribute to improving the reliability of pollen identification in both modern palynology and Quaternary palaeoecological reconstructions.

## 4. Materials and Methods

**Figure 4:**
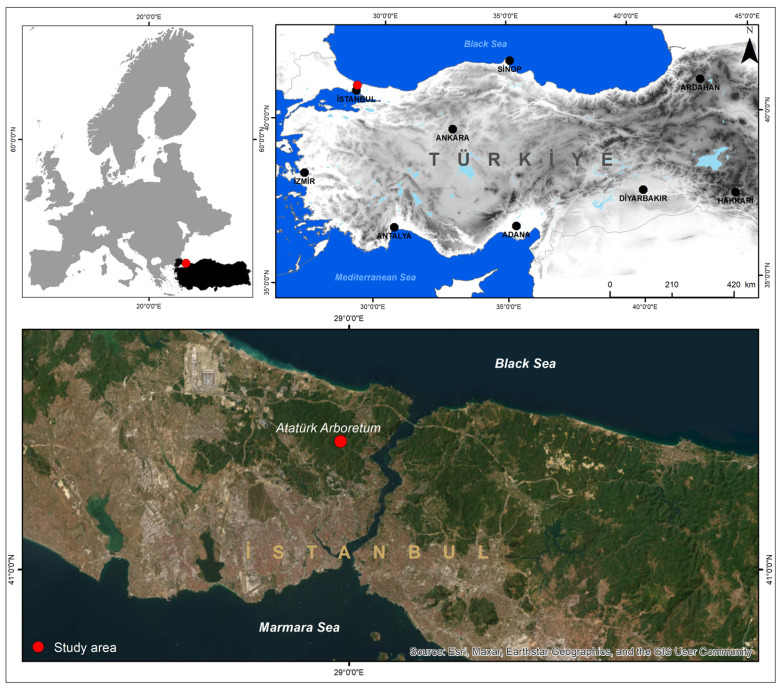
Location map of the study area.

Pollen grains of *Alnus glutinosa*, *Betula pendula*, *Carpinus betulus*, *Carpinus orientalis*, *Corylus avellana*, *Corylus colurna*, and *Ostrya carpinifolia*, which constitute the research material, were collected from the Atatürk Arboretum (Istanbul-Türkiye) by selecting mature male inflorescences during the pollination period (Figure 4).

The collected specimens were transferred to the Palynology Laboratory of the Faculty of Forestry, Istanbul University–Cerrahpaşa, where pollen slides were prepared using the method described by Wodehouse [8]. Measurements and microphotographs of pollen morphological characters were obtained using a Leica DM750 light microscope. The terminology proposed by Punt et al. [2], Hesse et al. [31], Beug [32], and Karlıoğlu Kılıç et al. [3] was used for the identification of pollen grains. SEM observations were carried out at the SEM Laboratory of the Faculty of Science, Istanbul University. Prior to SEM imaging, pollen grains were sputter-coated with platinum for 45 s using an EmiTech SC7620 (Istanbul/Türkiye) sputter coater. The coated specimens were examined and photographed with a JEOL NeoScope JCM-5000 benchtop SEM (Istanbul/Türkiye). Thirty measurements were taken under the light microscope for each morphological character, including Polar axis length (P), Equatorial diameter (E), pore length (plg), pore width (plt), and exine thickness (Ex).

All statistical analyses were performed to identify differences in pollen morphology among species. First, descriptive statistics (mean and standard deviation) were calculated for each species and each variable to assess intraspecific variation. One-way ANOVA was applied separately to each morphological variable to compare mean values among species. Tukey’s HSD post hoc test was conducted to determine pairwise differences among species for variables showing statistically significant differences in the ANOVA results. PCA was performed to evaluate the multivariate morphological structure and to reveal the combined contribution of all measured variables to species discrimination. All statistical analyses were carried out using the Python programming language (version 3.10; Python Software Foundation, Beaverton, OR, USA) [33]. Descriptive statistics were calculated using the pandas package package (version 2.0; pandas development team, Austin, TX, USA) [34], while one-way ANOVA and Tukey HSD tests were performed using the SciPy (version 1.11; SciPy community, USA) [35] and statsmodels (version 0.14; statsmodels developers, USA) [36] packages. PCA was conducted using the scikit-learn library (version 1.3; scikit-learn developers, Paris, France) [37]. Figures were generated using matplotlib (version 3.7; matplotlib development team, USA) [38].

## 5. Conclusions

This study demonstrates that pollen morphological differentiation within the Betulaceae is hierarchically structured and statistically robust rather than merely descriptive. By integrating detailed LM–SEM observations with ANOVA, Tukey’s HSD test and PCA, we identify pollen size as the dominant axis of interspecific variation, with aperture traits and exine thickness providing secondary and complementary resolution. The concordance between univariate and multivariate analyses confirms that interspecific variance significantly exceeds intraspecific variability, establishing a reproducible morphometric basis for species-level discrimination. Beyond validating classical palynological descriptions, this work introduces a quantitatively supported diagnostic hierarchy that reduces subjectivity and enhances taxonomic precision. The proposed framework offers a transferable model for other wind-pollinated woody taxa and strengthens the application of pollen morphology in both systematic botany and Quaternary paleoenvironmental research.

## Figures and Tables

**Table 1:** Taxonomic position and geographical distribution of the investigated Betulaceae taxa.

Species	Subfamily	General Distribution	Distribution in Türkiye	References
*Alnus* *glutinosa* (L.) Gaertn.	Betuloideae	NW Africa, Europe, Caucasus, Anatolia, N Iran	Northern Anatolia, also locally in Southern and Eastern Anatolia	[5]
*Betula* *pendula* Roth.	Betuloideae	Europe, Asia, Finland, Sweden, Norway, Central Europe	Northeastern and Eastern Anatolia	[5]
*Carpinus* *betulus* L.	Coryloideae	Europe, Türkiye, Caucasus, Iran	Widely distributed in the Black Sea Region; local populations in Amanos Mountains	[5]
*Carpinus* *orientalis* Mill.	Coryloideae	Balkans, Dalmatia, Italy, Sicily, Türkiye, Iran, Caucasus	Coastal areas, especially dry calcareous slopes at low elevations	[5]
*Corylus avellana* L.	Coryloideae	Europe, North Africa, Caucasus, W Asia	Throughout the Black Sea mountain range up to ~1800 m	[5]
*Corylus* *colurna* L.	Coryloideae	Southeastern Europe, Western Asia	Kazdağları, Bolu, Kastamonu, Ankara–Nallıhan, Zonguldak–Yenice, Rize, Trabzon	[5,6]
*Ostrya* *carpinifolia* Scop.	Coryloideae	Mediterranean region	Northern and southern Anatolia	[5,7]

**Table 2:** Summary of pollen morphological characteristics of the investigated Betulaceae taxa reported in previous studies.

Species	Pollen Size (µm)	Pore Number	Exine Thickness (µm)	References
*Alnus glutinosa*	P: 16–29.5; E: 23–31	3–7	0.75–2.0	[10,11,12,13,14,15,16,17,18]
*Betula pendula*	P: 16–35.51; E: 18.5–34	3	0.8–2.52	[12,13,19,20,21,22,23,24,25]
*Carpinus betulus*	P: 26–40; E: 26.34–46	3–5	0.7–1.53	[8,10,11,12,13,14,25,26,27]
*Carpinus orientalis*	P: 26.6–37.22; E: 26.79–28.84	3–5	0.87–1.75	[10,13,27]
*Corylus avellana*	P: 18–28; E: 22–34	3	1.09–1.5	[8,10,11,12,13,14,25,26,28]
*Corylus colurna*	P: 21.29–24.01; E: 29.26–33.47	3	1.1	[13,26]
*Ostrya carpinifolia*	P: 20–31; E: 19–28	3–4	0.89–1.5	[10,11,12,13,14,26]

**Table 3:** Measurement of pollen morphological characters.

Species	P (µm)	E (µm)	P/E	Plg (µm)	Plt (µm)	Ex (µm)	Number of Pores	Ornamentation	
								LM	SEM
*Alnus* *glutinosa*	17.53–24.71	20.96–29.50	0.88	3.05–4.22	1.27–2.85	1.03–1.65	5 (Stephanoporate)	Psilate	Microechinate
*Betula* *pendula*	20.64–24.82	23.27–26.96	0.89	2.58–3.51	2.03–3.24	0.63–1.16	3 (Triporate)	Psilate	Microechinate
*Carpinus betulus*	28.52–33.72	34.42–39.67	0.86	2.86–4.33	2.32–3.72	0.84–1.38	4–5 (Tetraporate, Stephanoporate)	Psilate	Microechinate
*Carpinus orientalis*	25.53–29.40	30–33.91	0.88	2.07–2.98	1.57–2.36	0.72–1.18	3 (Triporate)	Psilate	Microechinate
*Corylus avellana*	20.71–23.70	25.26–30.90	0.83	2.08–2.95	1.82–2.71	1.06–1.53	3 (Triporate)	Psilate	Microechinate
*Corylus* *colurna*	26.01–29.33	27.8–32.46	0.93	2.36–3.76	2.11–3.25	1.09–1.92	3 (Triporate)	Psilate	Microechinate
*Ostrya carpinifolia*	21.09–23.92	22.71–26.91	0.91	2.26–3.49	2.01–2.96	0.89–1.52	3 (Triporate)	Psilate	Microechinate

**Table 4:** Means and standard deviations of the species.

Species	P Mean	sd	E Mean	sd	plg Mean	sd	plt Mean	sd	Ex Mean	sd
*Alnus glutinosa*	21.77 ^a^	1.92	24.71 ^a^	2.13	3.50 ^b^	0.36	2.06 ^a^	0.41	1.27 ^b^	0.15
*Betula pendula*	22.64 ^a^	1.07	25.34 ^a^	0.83	3.06 ^b^	0.26	2.76 ^b^	0.30	0.86 ^a^	0.15
*Carpinus betulus*	31.35 ^d^	1.63	36.54 ^d^	1.55	3.47 ^b^	0.47	3.17 ^c^	0.43	1.04 ^ab^	0.13
*Carpinus orientalis*	27.62 ^c^	0.99	31.55 ^c^	1.00	2.59 ^a^	0.24	2.10 ^a^	0.22	0.94 ^a^	0.12
*Corylus avellana*	22.63 ^a^	0.80	27.03 ^b^	1.46	2.48 ^a^	0.24	2.21 ^a^	0.26	1.32 ^b^	0.15
*Corylus colurna*	28.16 ^c^	1.00	30.35 ^c^	1.29	2.99 ^ab^	0.36	2.76 ^b^	0.34	1.54 ^c^	0.21
*Ostrya carpinifolia*	22.62 ^a^	0.78	25.00 ^a^	1.37	3.05 ^b^	0.30	2.56 ^b^	0.32	1.21 ^b^	0.17

Note: Different letters within the same column indicate statistically significant differences among species according to Tukey’s HSD test (*p* < 0.05).

**Table 5:** ANOVA results.

Variable		
P	274.74	*p* < 0.001
E	283.16	*p* < 0.001
plg	42.02	*p* < 0.001
plt	45.59	*p* < 0.001
Ex	67.91	*p* < 0.001

**Table 6:** Diagnostic key to the investigated Betulaceae species based on pollen morphology.

Pollen ornamentation of all studied taxa is psilate under LM and microechinate under SEM:	
1a. Pollen grains 5-porate	*Alnus glutinosa*
1b. Pollen grains 3-porate or 4–5-porate	2
2a. Pollen grains 4–5-porate; P ≈ 31 µm; E ≈ 36 µm	*Carpinus betulus*
2b. Pollen grains strictly 3-porate	3
3a. Polar axis (P) > 27 µm	4
3b. Polar axis (P) < 27 µm	5
4a. P ≈ 28 µm; exine thickness ≈ 1.54 µm	*Corylus colurna*
4b. P ≈ 27 µm; pore length ≈ 2.59 µm	*Carpinus orientalis*
5a. Exine thickness < 1.0 µm	*Betula pendula*
5b. Exine thickness ≥ 1.0 µm	6
6a. Exine thickness ≈ 1.32 µm; E ≈ 27 µm	*Corylus avellana*
6b. Exine thickness ≈ 1.21 µm; E ≈ 25 µm	*Ostrya carpinifolia*

## Data Availability

All data are contained in the article.

## Abbreviations

The following abbreviations are used in this manuscript:

LM	Light Microscopy
SEM	Scanning Electron Microscopy
P	Polar axis length
E	Equatorial diameter
plg	Pore length
plt	Pore width
Ex	Exine thickness
ANOVA	ANalysis Of VAriance
Tukey’s HSD	Tukey’s Honestly Significant Difference
PCA	Principal Component Analysis
µm	Micrometre
